# Efficient state of charge estimation of lithium-ion batteries in electric vehicles using evolutionary intelligence-assisted GLA–CNN–Bi-LSTM deep learning model

**DOI:** 10.1016/j.heliyon.2024.e35183

**Published:** 2024-07-31

**Authors:** Muhammad Kamran Khan, Mohamad Abou Houran, Kimmo Kauhaniemi, Muhammad Hamza Zafar, Majad Mansoor, Saad Rashid

**Affiliations:** a School of Technology and Innovation, University of Vaasa, Finland; b School of Electrical Engineering, Xi'an Jiaotong University, No. 28, West Xianning Road, Xi'an, 710049, China; c Department of Engineering Sciences, University of Agder, NO-4879, Grimstad, Norway; d Department of Automation, University of Science and Technology of China, Hefei, China; e Department of Electrical Engineering, Hamdard University, Islamabad Campus, Islamabad, Pakistan

**Keywords:** Convolutional neural network (CNN), Bidirectional long short-term memory (bi-LSTM), Group learning algorithm (GLA), State of charge (SoC), Electric vehicles (EVs), Deep learning

## Abstract

The battery's performance heavily influences the safety, dependability, and operational efficiency of electric vehicles (EVs). This paper introduces an innovative hybrid deep learning architecture that dramatically enhances the estimation of the state of charge (SoC) of lithium-ion (Li-ion) batteries, crucial for efficient EV operation. Our model uniquely integrates a convolutional neural network (CNN) with bidirectional long short-term memory (Bi-LSTM), optimized through evolutionary intelligence, enabling an advanced level of precision in SoC estimation. A novel aspect of this work is the application of the Group Learning Algorithm (GLA) to tune the hyperparameters of the CNN–Bi-LSTM network meticulously. This approach not only refines the model's accuracy but also significantly enhances its efficiency by optimizing each parameter to best capture and integrate both spatial and temporal information from the battery data. This is in stark contrast to conventional models that typically focus on either spatial or temporal data, but not both effectively. The model's robustness is further demonstrated through its training across six diverse datasets that represent a range of EV discharge profiles, including the Highway Fuel Economy Test (HWFET), the US06 test, the Beijing Dynamic Stress Test (BJDST), the dynamic stress test (DST), the federal urban driving schedule (FUDS), and the urban development driving schedule (UDDS). These tests are crucial for ensuring that the model can perform under various real-world conditions. Experimentally, our hybrid model not only surpasses the performance of existing LSTM and CNN frameworks in tracking SoC estimation but also achieves an impressively quick convergence to true SoC values, maintaining an average root mean square error (RMSE) of less than 1 %. Furthermore, the experimental outcomes suggest that this new deep learning methodology outstrips conventional approaches in both convergence speed and estimation accuracy, thus promising to significantly enhance battery life and overall EV efficiency.

**Table:** Nomenclatures

**Acronyms**	
ANN	Artificial Neural Network
BJDST	Beijing Dynamic Stress Test
BiLSTM	Bidirectional Long Short-term Memory
BMS	Battery Management System
BSS	Battery Storage Systems
CNN	Convolutional Neural Network
DST	Dynamic Stress Test
EVs	Electric Vehicles
FUDS	Federal Urban Driving Schedule
GLA	Group Learning Algorithm
GCT	Granger Causality Test
HWFET	Highway Fuel Economy Test
LSTM	Long Short-term Memory
MAE	Mean Absolute Error
MAXE	Maximum Error
MSE	Mean Squared Error
NN	Neural Network
NRMSE	Normalized Root Means Square Error
RE	Relative Error
RMSE	Root Mean Square Error
RNN	Recurrent Neural Network
SoC	State of Charge
STC	Standard Test Conditions
UDDS	Urban Development Driving Schedule
**Variables &Symbols**	
$L_{b}$	Lower Limit
$U_{b}$	Upper Limit
$l_{r}$	Learning Rate
$N_{s}$	No. of LSTM units
$B_{n}$	No. of bi-directional layers
$I_{t}$	No. of iterations.
$n_{pop}$	New candidate solution
$c_{t}$	Cell state
$f_{g}$	Forget gate
$i_{g}$	Input gate
$o_{g}$	Output gate
${\vec{h}}_{t}$	Forward cell state of BiLSTM
${\overset{⃖}{h}}_{t}$	Reverse cell state of BiLSTM
$y_{t}$	Output of the LSTM and BiLSTM
$x_{t}$	Input to the LSTM and BiLSTM
LM	Long term memory (cell state) of LSTM
SM	Short term memory of LSTM
w,b	Weight and bias matrices
∂	Activation function
∗	Discrete convolution

## 1 Introduction

As the fuel crisis and environmental concerns continue to escalate, electric vehicles are gaining greater attention due to their clean, effective, and environmentally friendly features. Nevertheless, the efficacy of EVs significantly hinges on the performance of battery storage systems (BSS) concerning SoC assessment, fault diagnosis, and safety within a battery management system (BMS) environment [1]. Battery performance is a critical component in EVs that determines the vehicle's safety, reliability, and operating efficiency [2].

Li-ion batteries are increasingly becoming the primary power source for EVs due to their high energy and power density, fast charging, long lifespan, and eco-friendliness [3]. However, as the electric vehicle operating environment is complex and battery performance degrades over repeated use, a BMS is necessary to observe the battery's well-being. The BMS system ensures safe operation by guarding against overcharging and over-discharging the battery. The BMS system ensures safe operation by guarding against overcharging and over-discharging and thermal management of the battery [[4], [5], [6]]. Battery SoC gives a quick indication of how much charge is still left, and precise SoC calculation can help improve battery performance, lengthen battery life, and increase vehicle efficiency [7]. Therefore, SoC estimation of Li-ion batteries in EVs is important.

Currently, there are three key groups of SoC techniques. They are direct measurement methods, model-based, and data-driven strategies [8]. Some of the famous direct measurement techniques are open circuit voltage methods [[9], [10], [11]], current integration methods [12,13], and internal resistance methods [14,15]. Direct methods are considered simple, inexpensive, and give good real-time performance. However, the main disadvantages of these methods are time-consuming and not very accurate.

Model-based techniques employ mathematical models of batteries to determine the SoC accurately. These models consider different variables, such as battery chemistry, temperature, and aging. The internal states of the batteries are subsequently determined or estimated using adaptive filters and nonlinear estimation techniques. Various nonlinear state estimation algorithms and filters for SoC estimation are reported. Kalman filters, including different types, have been broadly used in the literature due to their robustness, accuracy, and ability to be easily implementable [16,17]. The quality of the battery models, which these model-based estimators are based on, has a considerable impact on their accuracy. However, these models typically rest on many fundamental presumptions that are imperative to make them controllable. Typical algorithms are discussed, including the Kalman filter [18,19], Fractional-order observer [20], proportional-integral observer [21], and sliding-mode observer [22]. However, model-based approaches are computationally intensive and require accurate battery models to achieve high precision.

When the mathematical model of a battery is either not known or the model failed to handle the unpredictability in the system, model-free machine learning methods are utilized [23]. Using a large amount of gathered data, machine learning approaches directly replicate the nonlinear correlations between the observed parameters and battery SoC. Artificial NNs [[24], [25], [26]], fuzzy logic (FL) [27], support vector machines [[28], [29], [30]], and Gaussian process regression (GPR) [31] are examples of machine learning techniques. Those techniques do not need a particular battery model, but the quantity and quality of the supplied training data have a significant impact on how well they estimate [29].

To estimate the Li-ion SoC, a Bayesian-optimized Bi-LSTM network is proposed to estimate the battery SoC by measuring different parameters like temperature, voltage, and current [32]. In Ref. [33], the authors used a gated recurrent unit (GRU) network for SoC approximation at different temperature ranges. In addition, its effectiveness was assessed using two popular Li-ion batteries. To address the nonparallel functionality seen in standard RNN algorithms, a combination of stacked Bi-LSTM and encoder-decoder Bi-LSTM mechanisms was presented in Ref. [34]. According to the authors, the HWFET condition showed a minimum mean absolute error (MAE) of 0.62 %. The study in Ref. [35] employed the particle swarm algorithm to optimize the hyperparameters parameters of a temporal convolutional network (TCN) for SoC estimation under varying temperature conditions. To improve the capture of time dependency in SoC estimation, an attention mechanism was integrated which enabled the network to focus on critical time steps. In Ref. [36], the authors used a hybrid CNN-LSTM network, where CNN was utilized to extract spatial information, and an LSTM network used historical inputs to retrieve temporal features. In Ref. [37], the LSTM recurrent neural network (LSTM-RNN), which uses extended input and limited output, was used to estimate battery SOC. The RMSE and maximum error (MAXE) of the experimental data were less than 1.3 % and 3.2 %, respectively. Table 1 shows a comparison of different research work on SoC Estimation.

**Table 1:** Detailed comparison with previous research work on SoC Estimation.

Ref	Year	Technique	Summary	Results
[38]	2023	ANA-LSTM	In this paper, an anti-noise adaptive long short-term memory neural network (ANA-LSTM) working with an adaptive feedback correction strategy is used to predict the remaining useful lift of Lithium-Ion batteries. A multi-stage fusion model for battery is considered which involves different parameters like Open Circuit Voltage (OCV), self-discharge resistance occurrence, polarization resistance and polarization capacitance. The results suggest that the proposed method for battery health prediction has better accuracy and least uncertainty when compared to other models.	RMSE = 0.60434 % MAE = 0.390774 % MAPE = 0.44672 %
[39]	2023	SF-GPR-LSTM	An improved singular filtering-Gaussian process regression-long term short-term memory (SF-GPR-LSTM) model is employed to predict the health of the battery for aerospace applications at low temperatures. The proposed technique has the advantage of comprehending the bidirectional information flow. The parameters like different temperatures, current amplification and stress are considered in the model. The SF-GPR-LSTM predicts the health of the battery with great accuracy and stability.	RMSE = 2.3484 % MAE = 0.82526 % MAPE = 0.90716 %
[37]	2023	LSTM-RNN	A Long term short-term based recurrent neural network (LSTM-RNN) model is used to predict the state of charge of lithium-ion battery using extended input (EI) and constrained output (CO). The extended input uses sliding window average voltage improves the mapping of different characteristics of the battery using LSTM-RNN model. At the output of LSTM-RNN, EI-based flow strategy is used to smooth output SOC of the proposed model. Using the same date sets, it was observed that EI-LSTM-CO has performed better for SOC estimation at unknown temperatures.	RMSE = 1.3 %
[40]	2024	PSO-TCN	A second order resistor-capacitance circuit based PSO-TCN model is proposed in this research for SOC estimation. In this model, open circuit voltages at different operating temperatures are used as an input for the TCN instead of terminal voltages. When the results of the generalized TCN model is compared with the proposed PSO-TCN model, it is observed that the proposed model greatly improves the accuracy and stability of the SOC estimation.	RMSE = 1.8 % MAXE = 7.4 %
[41]	2022	LPSO for BP Neural Networks	A PSO based on Levy flight strategy (LPSO) for BP neural networks is utilized for SOC estimation by considering voltage, current and temperature as input parameters. The predicted output of LPSO-BP algorithms is closest to the actual value when compared with the other methods validating its high accuracy and predictive ability.	MSE = 0.0116
[35]	2021	PSO-LSTM	A PSO-LSTM model is used to predict the values of SOC estimation and the results are compared with traditional LSTM model. Random noise is also introduced to enhance the computational abilities of the proposed model. The SOC estimation provided by the proposed method is satisfactory.	RMSE = 0.4540 % MAE = 0.3493 %
[42]	2021	CWRNN	In order to improve the performance of recurrent neural networks (RNN), clockwork RNN (CWRNN) is proposed in this literature. The proposed method divides hidden layers into large and small modules enabling them to capture small changes more quickly thus reducing the computation cost at varying temperatures.	MAE = 1.29 %
[43]	2023	Variational EKF	In order to improve the estimation accuracy and robustness, a novel variational extended Kalman filter based on least square is suggested which uses a second-order equivalent circuit model of the battery. The modelling error was decreased by identifying system parameters online. The results proved the better performance of variational EKF as compared to traditional EKF.	MSE = 0.0019 MAE = 0.0343
[44]	2022	BLS-LSTM	To predict the remaining useful life (RUL) of Lithium-ion battery, Broad Learning System (BLS) algorithm is combined with Long-term short-term neural network, a fusion neural network is proposed. BLS is used creates feature nodes which are used as the input layer LSTM neural network The results prove that the proposed method predicts the battery capacity precisely.	RMSE = 0.096 RMSE = 0087
[45]	2024	Bidirectional LSTM-Reduction	A Bidirectional Long Short-Term Memory Reduction (LSTM-Reduction) model is used for State of Health (SOH) estimation. This model incorporates both forward and reverse LSTM structures which help in filtering nonessential data providing reliable results related to SOH estimation. The results predicted turn out to be highly accurate when compared to actual values.	MAE <0.25 % RMSE<0.32 % $R^{2}>$ 0.96

**Fig. 1:**
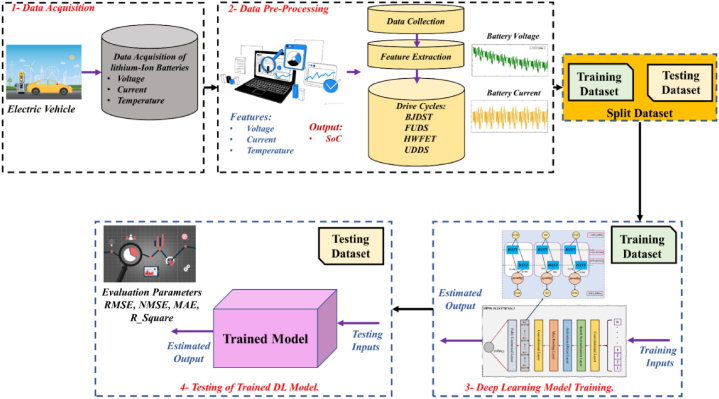
Proposed SoC Estimation Process for Li-ion batteries used in EVs.

The primary flaw in the machine learning-based approaches is the requirement to artificially configure the hyperparameters of the network, which leads to high unpredictability and significantly reduces the predictive power of the model. To overcome this issue, the GLA algorithm is applied to tune the hyperparameters of the deep Bi-LSTM, which enhances the stability and prediction ability of LSTM. The proposed network is trained using six different datasets of EV discharge profiles. They are the Highway Fuel Economy Test (HWFET), the US06 test, Beijing Dynamic Stress Test (BJDST), the dynamic stress test (DST), the federal urban driving schedule (FUDS), and the urban development driving schedule (UDDS). Detailed procedures of the proposed SoC estimation process are displayed in Fig. 1.

The main contributions of this work are summarized as follows.

• A novel hybrid architecture is proposed that combines the strengths of convolutional neural networks (CNNs) and bidirectional long short-term memory networks (Bi-LSTMs). This integrated model harnesses CNNs to extract spatial features from data effectively, which are crucial for understanding the structural characteristics of input data. Concurrently, Bi-LSTMs are utilized to capture temporal dependencies, allowing the model to monitor and predict the battery's SoC over time based on past and present data. This synergy enhances the accuracy and efficiency of SoC estimations, making it a robust tool for managing the performance and longevity of Li-ion batteries in EVs.
• The hyperparameters of the CNN–Bi-LSTM network are finely tuned using the Group Learning Algorithm (GLA), a sophisticated approach that enhances model training by finding the optimal configuration settings efficiently. GLA applies principles of evolutionary computation and group dynamics to optimize the learning process, significantly improving the predictability and stability of the model. This method not only facilitates faster convergence but also enhances the model's adaptability to various operational conditions.
• A comprehensive performance analysis is conducted to benchmark the proposed GLA–CNN–Bi-LSTM model against two other models: a GLA-based LSTM model and a GLA-based CNN model. This comparative study highlights the individual and collective strengths of integrating CNNs with Bi-LSTMs, compared to using each model independently. The evaluation includes various metrics to rigorously assess and demonstrate the superior capabilities of the hybrid model in real-world scenarios, providing a transparent and empirical basis for the model's efficacy.
• To ensure the robustness and practical applicability of the proposed model, it is tested against six different drive cycle tests commonly used in the industry. These include the Highway Fuel Economy Test (HWFET), Beijing Dynamic Stress Test (BJDST), Federal Urban Driving Schedule (FUDS), Dynamic Stress Test (DST), Urban Development Driving Schedule (UDDS), and US06. These tests simulate a wide range of driving conditions, from urban traffic patterns to highway speeds, providing a comprehensive set of scenarios to validate the model's performance. This extensive testing confirms the model's ability to accurately predict SoC under diverse conditions, enhancing its utility for real-world applications.

This work is organized as follows. Section 2 presents the work methodology, including GLA, CNN, and LSTM models. Section 3 gives details about Bi-LSTM, GLA-Bi-LSTM models, and the proposed GLA–CNN–Bi-LSTM Model. In addition, the experimental setup is described. Section 4 presents the results and discussions. Finally, section 5 concludes the work.

## 2 Methodology

### 2.1 Modelling of GLA

The group learning algorithm is inspired by the interactions between individuals in a group and the influence of a group leader on their members [46]. The algorithm assumes that the population consists of individuals who are employees of an organization. Every organization has a manager who is the individual with the best fitness. The population is then divided into a predetermined number of groups, and the fittest candidate within each group is chosen as the group leader. The concept of having a manager and group leaders in GLA is to enhance the performance of individuals within the groups and improve their overall fitness. GLA balances local and global search to explore the search space effectively and improve overall fitness.

i. Initialization

The GLA is initialized using equation (1).

(1) $$pop=rand\times \left(U_{b}\;-\;L_{b}\right)+L_{b}$$

where $L_{b}$, $U_{b}$ and rand represent the lower limit, upper limit, and randomly generated value. After the population is initialized, it is divided into groups of *n* individuals. Within each group, the individual with the highest fitness level is chosen to be the group leader. This selection process involves identifying the fittest individuals in each group and assigning them the positions of group leaders.

ii. Effect of the manager on group leader

Equation (2) shows the effect of the manager on the group leader.

(2) NGL=(gl−m)*rn

where NGL is new group leader after the manager influence, gl is the current group leader, m is the manager having best fitness in the population, and rn is a random number between 0 and 1.

iii. Effect of the group leader on individuals

Equation (3) is used to demonstrate the effect of the group leader on individuals mathematically.

(3) $$n_{can}\left(i\right)=\left(NGL-can\left(i\right)\right)*rn$$

where $n_{can}$ is the new candidate solution after influenced by the NGL. can(i) is individual inside the group, and rn is a random number.

iv. Effect of the manager on individuals:

The effect of the manager on the individuals within the groups is given by equation (4).

(4) $$n_{pop}\left(i\right)=\left(can\left(i\right)-m\right)*rn$$

where $n_{pop}$ is the new candidate solution after it is affected by the manager, can(i) is individual inside the group, and r is a random number in the range [0, 1].

In addition, there may be some uncontrolled randoms factors outside the group that affect the performance of individuals within the group. This effect is modelled by randomly altering positions of some of the individuals by utilizing a mutation operator.

### 2.2 One-dimensional convolutional neural network (1D CNN)

1D CNN is a neuron-based network designed for handling one-dimensional sequences of time series or text-based data. It is different from a 2D CNN in which filters move across both spatial dimensions of an image because, in 1D CNN, the layer filters move in only one dimension [47]. The architecture of a 1D CNN includes one or more convolutional layers, accompanied by one or more fully connected layers. Convolutional layers use filters to draw out features from the input sequence and create feature maps. These filters slide over the input sequence, calculating the dot product of the filter weights and the input at each position and outputting a feature map, as represented by equation (5).

(5) $$h_{k}=\partial \left(w_{k}*x_{k}+b_{c}\right)$$

where the symbol ∗ indicates the discrete convolution of the filter weight ${\;w}_{k}$ and input signal *x*_*k*_. $b_{c}$ is a bias parameter, which shall be learned during the training process. ∂ is the underlying activation function. The feature maps are then passed through a pooling layer to downsample and reduce the spatial size of the feature maps.

### 2.3 LSTM model

**Fig. 2:**
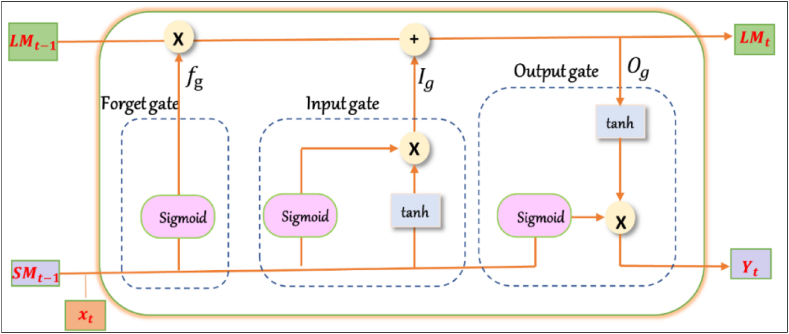
Structure of LSTM model.

An LSTM neural network is a kind of RNN that can learn long-term dependencies in sequential data. It was developed to solve the vanishing gradient problem in traditional RNNs, which can make it difficult for the model to learn long-term dependencies [48]. LSTMs have a similar structure to RNNs but include specialized memory cells and gating mechanisms. The memory cells allow the network to store and access information selectively over long periods. The memory cell is controlled by an output gate, an input gate, and a forget gate. All the three gates control the flow of information that goes in and out of the memory cell, which allows the network to remember or forget information as necessary. The LSTM architecture is illustrated in Fig. 2. Equations (6), (7), (8), (9) clarify how the internal unit of an LSTM is updated.

(6) $$f_{g}=\sigma \left(w_{f}x_{t}+w_{fh}{SM}_{t-1}+b_{f}\right)$$

(7) $$i_{g}=\sigma \left(w_{ix}x_{t}+w_{ih}{SM}_{t-1}+b_{i}\right)$$

(8) $$o_{g}=\sigma \left(w_{o}x_{t}+w_{oh}{SM}_{t-1}+b_{o}\right)$$

(9) $$s_{t}=\tan\;h\left(w_{c}x_{t}+w_{ch}{SM}_{t-1}+b_{o}\right)$$

where w is the weight matrices, b is deviation vectors of the three gates, σ is sigmoid activation function, tanh is layer that updates the internal cells and cell outputs by normalizing the data to lie between (−1,1), and the $s_{t}$ is a vector of potential values after the tanh layer. The cell state ${LM}_{t}$ and the output $y_{t}$ are represented by equations (10), (11).

(10) $${LM}_{t}=f_{g}{LM}_{t-1}+i_{g}s_{t}$$

(11) $$y_{t}=o_{g}+\tanh\;\left({\text{LM}}_{t}\right)$$

## 3 Proposed GLA–CNN–Bi-LSTM model and experimental setup

### 3.1 BiLSTM model and hyperparameters

**Fig. 3:**
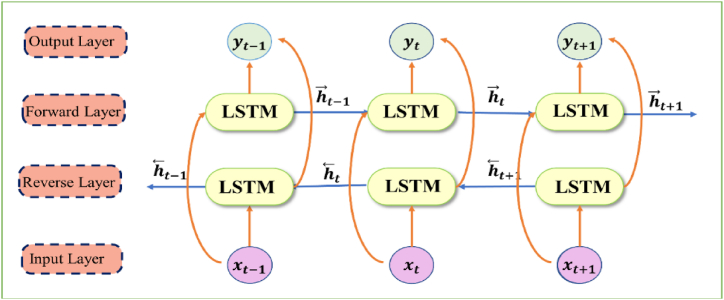
Structure of BiLSTM network.

The LSTM design has a restriction where it can solely capture positive dependencies, and as a result, it may discard crucial information via the long-term gated memory. To overcome this shortcoming, researchers introduced the Bidirectional Long Short-Term Memory (BiLSTM) neural network that enables the network to process the backward and forward flow of information. BiLSTM incorporates a pair of hidden layers that handle input data in both directions, as shown in Fig. 3. The output layer receives these hidden sequences and combines them. BiLSTM employs forward and backward sequences to upgrade the output sequence with the help of equation (12) and equation (13).

(12) $${\vec{h}}_{t}=f_{lstm}\left(x_{t},{\vec{h}}_{t-1}\right)$$

(13) $${\overset{⃖}{h}}_{t}=f_{lstm}\left(x_{t},{\overset{⃖}{h}}_{t+1}\right)$$

where ${\vec{h}}_{t},{\overset{⃖}{h}}_{t}$ represent forward and reverse cell states at time t. $h_{t-1},h_{t+1}$ shows past and future cell states and $x_{t}$ represents the input to LSTM cell at time t. After the two LSTM layers process the input data $x_{t}$, their outputs are combined by concatenating them, and the resulting sequence is then fed through a final output layer to generate the ultimate output $y_{t}$, as shown in equation (14), where $w_{y}$ and $b_{y}$ represent weight and bias matrix.

(14) $$y_{t}=w_{y}{\vec{h}}_{t}+w_{y}{\overset{⃖}{h}}_{t}+b_{y}$$

Aligning the structure of the network model with the input data, enhance the training speed, and boost the overall network performance, determining the optimal parameters of BiLSTM has become a new challenge. Some of the hyperparameters in the BiLSTM model include the learning rate, number of LSTM units, number of bidirectional layers, and number of iterations. If the number of LSTM units and bidirectional layers are too small, it may not be possible to capture all the information needed for the training process of the samples. If the number of samples is too large, it may lead to overfitting. The number of iterations means the number of times the training data is processed by the network. If this value is too large, the convergence rate may be too slow, whereas if it is too small, the model may not converge. The learning rate determines the size of the step during the optimization process. Large or small learning rates may result in overshooting the optimal value or slow convergence, respectively.

### 3.2 GLA-based tuning of BiLSTM

The performance of SoC estimation using currently available data-driven algorithms is typically assessed by adjusting various hyperparameters. However, this approach can be laborious and time-consuming, requiring significant human effort to achieve satisfactory results. To overcome this challenge, an alternative solution can be to make an improved SOC estimation mechanism that can intelligently calculate SoC considering different operating conditions. One productive strategy is achieved by combining data-driven algorithms with heuristic optimization techniques to produce an intelligent hybridized optimization approach. This optimized data driven SoC estimation could be designed by improving the hyperparameters to achieve the minimized value of the objective function while keeping in check all the constraints. The hyperparameter space of BiLSTM is extensive and difficult to traverse comprehensively. Therefore, a highly efficient GLA algorithm can be used to obtain well-performing hyperparameters. The parameters of the BiLSTM model are the learning rate, number of LSTM units, number of bidirectional layers, and number of iterations. The range of values for these parameters is set as follows: learning rate ($l_{r}$) is 0.0001–0.1, the number of LSTM units ($N_{s}$) is 5–50, the number of bidirectional layers ($B_{n}$) is 3–30 and the number of iterations is $(I_{t}$) 50 to 500.

### 3.3 GLA–CNN–Bi-LSTM model for SoC estimation

To accurately estimate battery SoC, the spatial and temporal features must be considered. Spatial features mean the interrelationships between the current inputs while temporal features point to the correlations between the present SoC and inputs of the past. To address this challenge, a hybridized CNN–Bi-LSTM network is proposed to handle both spatial and temporal features for relatively accurate and sturdy SoC estimation. In order to draw out the advanced spatial features present in the original date, CNN is executed while the LSTM is utilized to model the interrelationship between the current SoC and historical inputs.

**Fig. 4:**
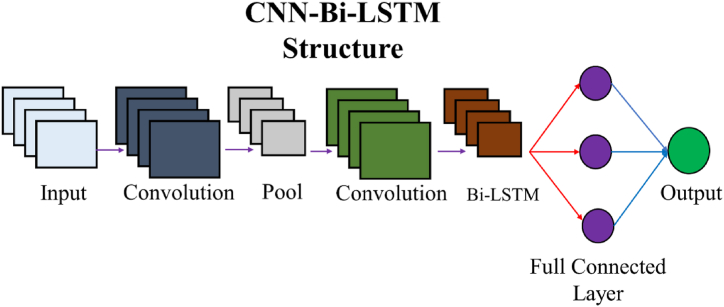
Hybrid CNN–Bi-LSTM structure.

The 1D convolutional layer plays an important role in extracting data features that can be used as an input for the LSTM layer. By adjusting the weight of the convolution kernel and the width of the window, multiple data features can be drawn out to improve the network's performance. The LSTM considers the correlations between the present and past inputs while CNN restricts the network to consider the relationships between the present inputs. Fig. 4 displays the hybrid CNN-LSTM model.

**Fig. 5:**
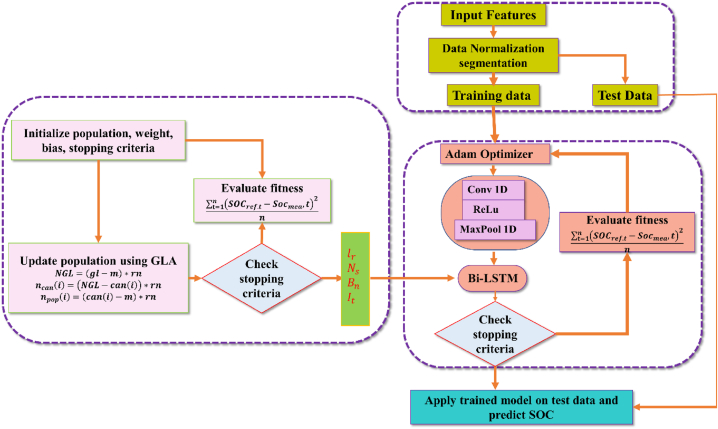
GLA–CNN–Bi-LSTM flowchart.

The MSE loss function is utilized to train the deep neural network. Within the training process, the overall loss function is calculated at the end of each forward pass, using MSE.

(15) $$cost=\frac{\sum_{t=1}^{n}{\left({SOC}_{ref.t}-{Soc}_{mea},t\right)}^{2}}{n}$$

where ${SOC}_{ref.t}$ is the true SoC value while ${Soc}_{mea},t$ is the output of the proposed network at time *t*. The proposed GLA–CNN–Bi-LSTM model is presented in Fig. 5. The performance of the suggested network is assessed during testing using the RMSE, $R^{2}$, and MAE.

(16) $$RMSE=\sqrt{\frac{\sum_{t=1}^{n}{\left(y_{t}-p_{t}\right)}^{2}}{n}}$$

(17) $$RE=\sum_{t=1}^{n}\left|\frac{y_{t}-p_{t}}{y_{t}}\right|$$

(18) $$R^{2}=1-\;\frac{\sum_{t=1}^{n}{\left(y_{t}-p_{t}\right)}^{2}}{\sum_{t=1}^{n}{\left(y_{t}-\bar{y_{j}}\right)}^{2}}$$

where $y_{t}$ and $p_{t}$ represent the true and predicted values. $\bar{y_{t}}$ is the mean value and *n* being the number of samples. While higher R^2^ values imply better accuracy. In addition, higher RE and RMSE values imply below average prediction accuracy. Table 2 below lists the GLA–CNN–Bi-LSTM Model's optimized hyperparameters.

**Table 2:** Optimized hyperparameters of GLA–CNN–Bi-LSTM model.

Hyperparameters	Values
**No. of Hidden Neurons**	8
**No. of bidirectional Layers**	4
**Optimization Algorithm**	GLA (Group learning Algorithm)
**Learning rate**	0.0015
**Activation Function for Output Layer**	Sigmoid
**Number of iterations**	65

### 3.4 Experimental setup

The AMD Ryzen 5 5500U with Radeon Graphics 2.10 GHz hardware was used in this work, and MATLAB 2022 was used for SoC estimate. R-Square (*R*^2^), relative error (RE), normalized root means square error (NRMSE), RMSE), and MAE metrics are used to assess the effectiveness of the proposed GLA–CNN–Bi-LSTM model in forecasting the SoC on various drive cycles [37]. The used drive cycles include HWFET, the US06 test, BJDST, DST, FUDS, and UDDS.

A. Dataset for SoC

We used data from the CALCE Research Group that were made available to us for this investigation [49]. The used batteries are cylindrical INR21700-40T Li-ion. The data was gathered over a variety of driving cycles, following industry-standard charging and discharging procedures. The cell was discharged at three different temperatures (25 °C, 0 °C, and 45 °C) after being charged using a constant current/constant voltage methodology. Table 3 contains the battery specs that were employed in the study.

**Table 3:** Battery Specs used in Experimental Setup for Generation of Different Datasets.B.Data Diversity

Type	Capacity (Ah)	Voltage (V)	Rated Current (A)	Cut off Voltage.	Operating temperature
INR21700-40T	4.0	3.60	35	2.4/4.2	0–45^0^C

**Fig. 6:**
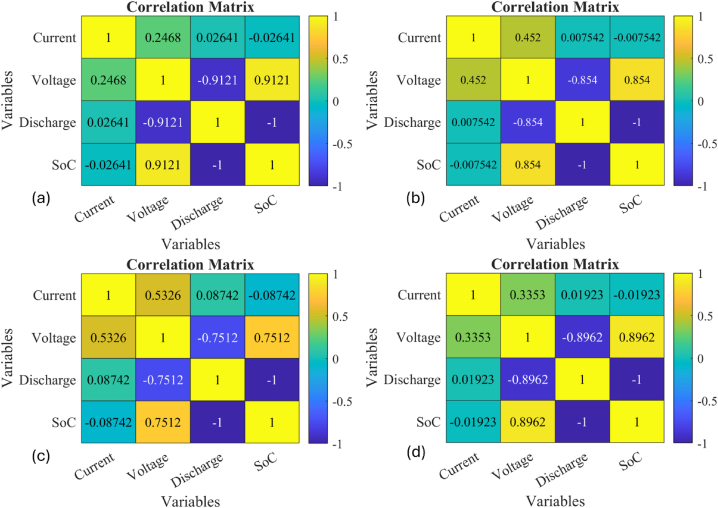
(a–d). The data correlation Matrix at STC conditions for 4-main data clusters for SoC with respect to voltage, current and Discharge. (a) BJDST. (b) DST. (c) FUDS. (d) US06.

There are multiple highly effective datasets to generate an excellent training environment [50]. Ten distinct cases for data are utilized. The correlation matrix (CM) is employed to show the relationship between several variables to highlight the strong association between multiple variables with the strength and direction of the relationships. Each cell in the matrix contains a correlation coefficient, ranging from −1 to 1. When the correlation coefficient is 1, it means that as one variable rises, the other variable also increases. When two variables have a correlation value of −1, they are strongly negatively correlated, indicating that when a variable rises, the other falls. There is no connection between the variables when the correlation coefficient is 0. CM advocates for the identification of patterns and trends in the data while identifying multicollinearity in multiple regression models. The analysis shows a strong positive relationship between SoC with voltage and discharge capacity, as compared to current density. In addition, urban settings (BJDST and US06) affect the SoC more comparatively. Lower temperatures, repeated discharging, and charging also impact the SoC in the long run. Fig. 6(a–d) presents the strong diversity of BJDST, DST, FUDS and US06 datasets at STC conditions.

## 4 Results and discussions

In this section, the suggested architecture is trained to calculate the battery SoC at various temperatures to account for the impacts of ambient temperature. The GLA–CNN–Bi-LSTM model is trained offline using data gathered at 0 °C, 25 °C, and 45 °C.

### 4.1 Evaluation at 0 degrees Celsius

**Fig. 7:**
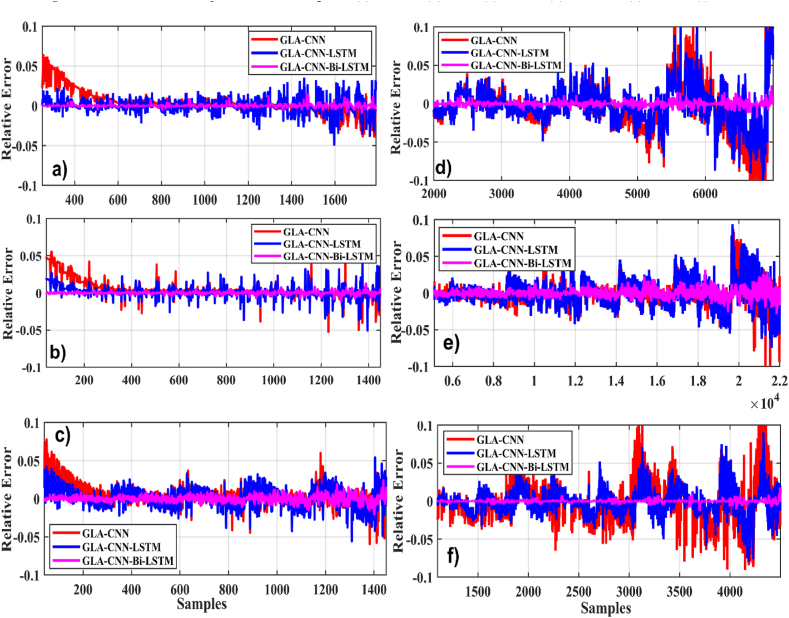
Relative error comparison at zero degrees. (a) BJDST. (b) DST. (c) FUDS. (d) HWFET. (e) UDDS. (f) US06.

In the first case, we evaluated the proposed technique GLA–CNN–Bi-LSTM, and other approaches like CNN, CNN-LSTM, GLA-LSTM, GLA-CNN. Various regression metrics, such as RMSE, MAE, RE, and NMSE are considered. for a temperature of 0 °C with six different drive cycles. The findings demonstrate that the suggested methodology is more precise and stable than the existing SoC estimating techniques. The results are presented in Table 4. For example, when tested using BJDST, it is concluded that the GLA–CNN–Bi-LSTM technique performs significantly better than GLA-CNN and GLA-LSTM since it has the lowest values of RMSE (1.4952e-03), NMSE (3.2346e-03), MAE (0.0026), and RE (0.0061). Fig. 7(a–f) displays the relative error comparison of the GLA–CNN–Bi-LSTM model (in red) with CNN and CNN-LSTM for various datasets at zero degrees. Results show that GLA–CNN–Bi-LSTM technique performs significantly better than GLA-CNN and GLA-LSTM for all considered datasets.

**Table 4:** SoC estimation evaluation at zero-degree datasets of drive cycles.

Drive Cycle	Technique	RMSE	NMSE	MAE	R^2^	RE
**BJDST** (Beijing Dynamic Stress Test)	GLA–CNN–Bi-LSTM	**1.4952e-03**	**3.2346e-03**	**0.0026**	**99.22**	**0.0061**
	GLA–CNN–LSTM	8.6727e-03	2.0975e-02	0.0126	97.83	0.0090
	GLA-CNN	2.5371e-01	0.0189	0.5619	93.65	0.3951
**DST** (Dynamic Stress Test)	GLA–CNN–Bi-LSTM	**1.7494e-03**	**1.8906e-03**	**2.8229e-03**	**99.57**	**0.0115**
	GLA–CNN–LSTM	6.1198e-03	7.0523e-03	0.0056	98.08	0.0389
	GLA-CNN	2.7843e-02	0.0251	0.0731	95.78	0.0802
**FUDS** (Federal Urban Driving Schedule)	GLA–CNN–Bi-LSTM	**1.5944e-03**	**1.3310e-03**	**0.0014**	**99.18**	**0.0012**
	GLA–CNN–LSTM	8.6931e-03	9.6094e-03	0.0165	95.76	0.0201
	GLA-CNN	2.6602e-02	0.0247	0.0174	92.30	0.0416
**HWFET** (Highway Fuel Economy Test)	GLA–CNN–Bi-LSTM	**1.1366e-03**	**1.3597e-03**	**0.0022**	**99.03**	**0.0083**
	GLA–CNN–LSTM	9.2804e-03	9.0643e-03	0.0624	97.09	0.0224
	GLA-CNN	4.3533e-02	0.0191	0.1079	94.58	0.0344
**UDDS** (Urban Development Driving Schedule)	GLA–CNN–Bi-LSTM	**4.8454e-03**	**3.1716e-03**	**0.0176**	**99.05**	**0.0017**
	GLA–CNN–LSTM	4.0889e-02	5.4704e-02	0.1692	96.61	0.0221
	GLA-CNN	8.5446e-02	0.0905	0.2969	94.54	0.0406
**US06**	GLA–CNN–Bi-LSTM	**2.3408e-03**	**4.7569e-03**	**1.6827e-03**	**99.53**	**0.00145**
	GLA–CNN–LSTM	9.3589e-03	1.9891e-02	0.0172	96.67	0.0169
	GLA-CNN	3.5509e-02	0.0161	0.6652	94.81	0.0681

Furthermore, when R-Square is considered, its maximum value is 99.22, which is the closest to 100, shows that GLA–CNN–Bi-LSTM accurately predicts the value of SoC. The same holds true when DST is considered. When compared to GLA-CNN and GLA-LSTM, GLA–CNN–Bi-LSTM has the lowest values of RMSE (1.7494e-03), NMSE (1.8906e-03), MAE (2.8229e-03), and RE (0.0115), while GLA-LSTM performs somewhat better than GLA-CNN. When the suggested technique GLA–CNN–Bi-LSTM is employed for the prediction of SoC, three additional tests, namely the HWFET, UDDS, and USO6, all yield encouraging results. The R-Square scores for each stress test demonstrate that GLA–CNN–BiLSTM provided the most precise forecast of battery SoC. The accuracy of the projected values by GLA–CNN–BiLSTM was also indicated by the reduced values of RMSE, NMSE, MAE, and RE.

**Fig. 8:**
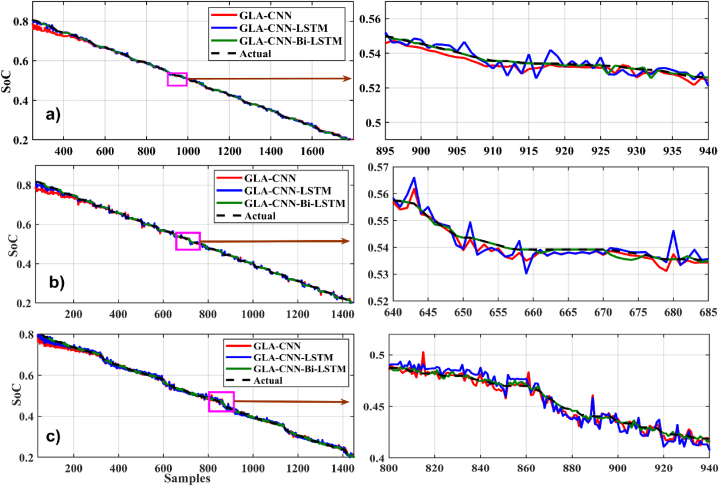
SoC estimation comparison at zero degrees. (a) BJDST. (b) DST. (c) FUDS.

**Fig. 9:**
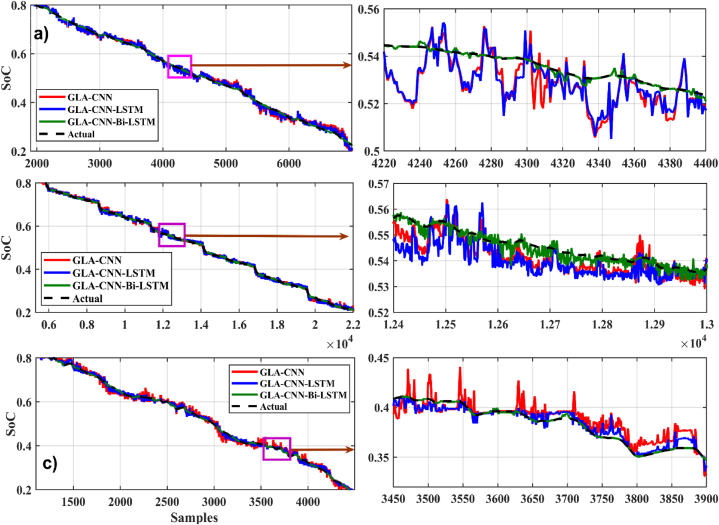
SoC estimation comparison at zero degrees. (a) HWFET. (b) UDDS. (c) US06.

In Fig. 8, Fig. 9, different graphs are plotted between time samples along the x-axis and SoC along the y-axis at 0 °C. For the loading profile BJDST at 0 °C, as shown in Fig. 8 (a), the performance of GLA–CNN–Bi-LSTM (in red) is better than that of the other two networks because the response has very few fluctuations. As can be seen, the GLA–CNN–LSTM-based network results in the greatest number of fluctuations in SoC estimation, while the performance of

GLA-CNN is better than GLA–CNN–LSTM, it is still worse than GLA–CNN–Bi-LSTM. When the loading profile is switched from BJDST to DST, keeping the same temperature, almost similar behavior is observed as shown in Fig. 8 (b). The response of GLA–CNN–Bi-LSTM is very smooth as compared to GLA-CNN and GLA–CNN–LSTM. Compared to the previous loading profile, there is a significant increase in ripples. In addition, when considering the loading profiles FUDS, HWFET, UDDS and US06, it is observed that the performance of GLA–CNN–Bi-LSTM is still superior to CLA-CNN and GLA–CNN–LSTM as represented by Fig. 8, Fig. 9 respectively.

### 4.2 Evaluation at 25 degrees Celsius

**Fig. 10:**
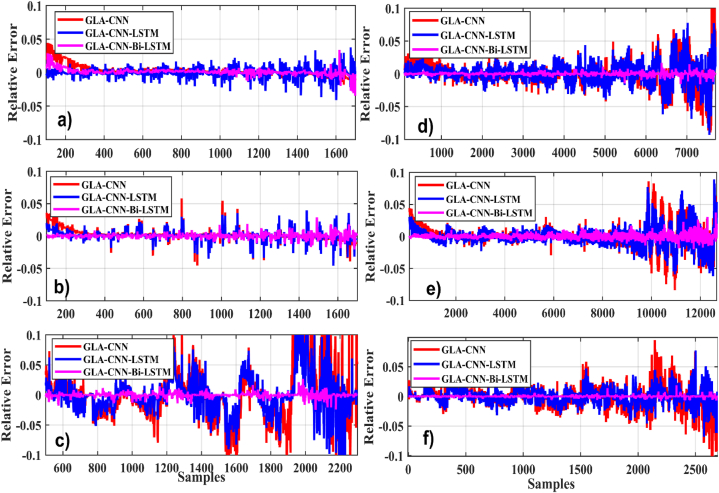
Relative error comparison for 25-degree. (a) BJDST. (b) DST. (c) FUDS. (d) HWFET. (e) UDDS. (f) US06.

Table 5 displays the values of various GLA–CNN–Bi-LSTM, GLA-LSTM, and GLA-CNN parameters when a temperature of 25 °C is considered and SoC is measured after the battery is discharged under different drive cycles. When BJDST is considered, the values of all the parameters are the lowest, and the R-Square value is the highest. This demonstrates that the prediction ability of the GLA–CNN–Bi-LSTM technique is superior to other models. In addition, the R-Square value for the suggested technique is the maximum for the remaining five drive cycles used for simulation while all the other parameters have minimal values for the proposed model. Moreover, compared to GLA-CNN and GLA-LSTM, GLA–CNN–Bi-LSTM has low prediction error, as indicated by the relative error comparisons in Fig. 10(a–f).

**Table 5:** SoC estimation at 25° datasets of drive cycles.

Drive Cycle	Technique	RMSE	NMSE	MAE	R^2^	RE
**BJDST**	GLA–CNN–Bi-LSTM	**2.9560e-03**	**1.4256e-03**	**0.0010**	**99.06**	**1.2370e-03**
	GLA–CNN–LSTM	9.1887e-03	8.6789e-03	0.0061	97.19	0.0237
	GLA-CNN	2.3317e-02	0.0221	0.0917	94.30	0.0406
**DST**	GLA–CNN–Bi-LSTM	**3.3190e-03**	**4.0640e-03**	**0.0024**	**99.12**	**0.0015**
	GLA–CNN–LSTM	8.250e-3	0.0005	0.0025	96.88	0.0212
	GLA-CNN	2.2283e-2	0.0018	0.0056	94.75	0.0348
**FUDS**	GLA–CNN–Bi-LSTM	**3.073e-03**	**2.9436e-03**	**0.0014**	**98.79**	**0.0043**
	GLA–CNN–LSTM	2.1129e-2	1.391e-2	0.0398	96.35	0.0103
	GLA-CNN	5.386e-2	6.574e-2	0.0634	95.11	0.0870
**HWFET**	GLA–CNN–Bi-LSTM	**1.764e-3**	**1.6334e-3**	**0.0019**	**99.11**	**0.0038**
	GLA–CNN–LSTM	5.875e-3	8.748e-3	0.0438	97.42	0.0255
	GLA-CNN	2.778e-2	6.569e-2	0.0781	95.33	0.0706
**UDDS**	GLA–CNN–Bi-LSTM	**1.095e-3**	**2.241e-3**	**0.0124**	**98.56**	**0.0075**
	GLA–CNN–LSTM	9.714e-3	4.152e-2	0.1020	96.78	0.0379
	GLA-CNN	6.433e-2	7.731e-2	0.0155	94.36	0.0439
**US06**	GLA–CNN–Bi-LSTM	**1.054e-3**	**1.436e-3**	**0.0001**	**99.17**	**0.0059**
	GLA–CNN–LSTM	9.711e-3	7.760e-3	0.0094	96.99	0.0463
	GLA-CNN	4.470e-2	8.609e-2	0.0178	94.56	0.1603

**Fig. 11:**
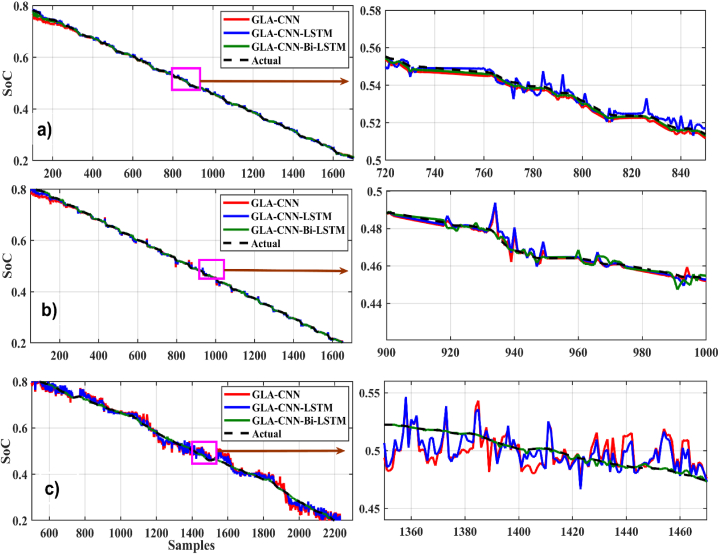
SoC estimation comparison at 25°. (a) BJDST. (b) DST. (c) FUDS.

**Fig. 12:**
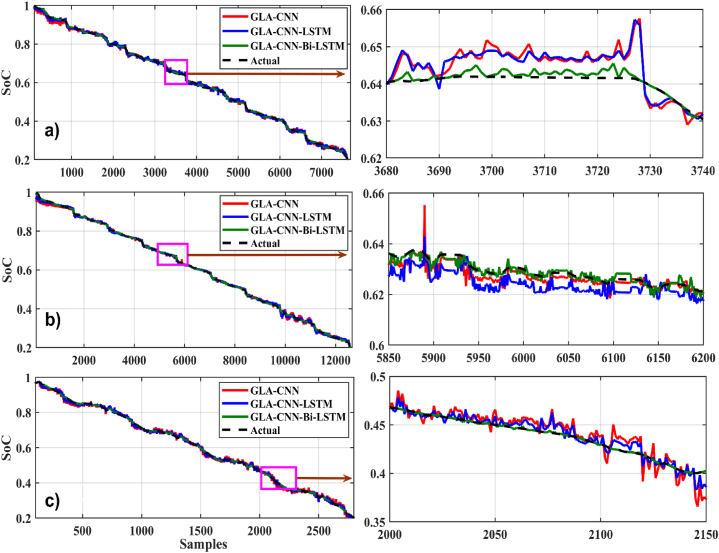
SoC estimation comparison at 25°. (a) HWFET. (b) UDDS. (c) US06.

Fig. 11, Fig. 12 present the SoC estimation comparison at 25°. For the loading profile BJDST at 25 °C, as shown in Fig. 11(a), the highlighted sample demonstrates how much smoother the values predicted by the suggested method are. When DST and FUDS are used as shown in Fig. 11(b) and (c), the GLA–CNN–Bi-LSTM projected values are less smooth than they were in the case of BJDST, but the values are still considerably close to the real values when compared with other models. The results in Fig. 11, Fig. 12 clearly show that the proposed algorithm outperforms other algorithms in terms of estimation accuracy. For example, Fig. 12(a and b and c) shows the relationship between HWFET, UDDS, US06, and SoC considering various prediction approaches. The values predicted by the GLA–CNN–Bi-LSTM model look largely flat and smooth with only a few ripples at specific intervals. However, using the other models, there is a distinct rise in the roughness of the predicted values by GLA-CNN and GLA–CNN–LSTM.

### 4.3 Evaluation at 45 degree Celsius

**Fig. 13:**
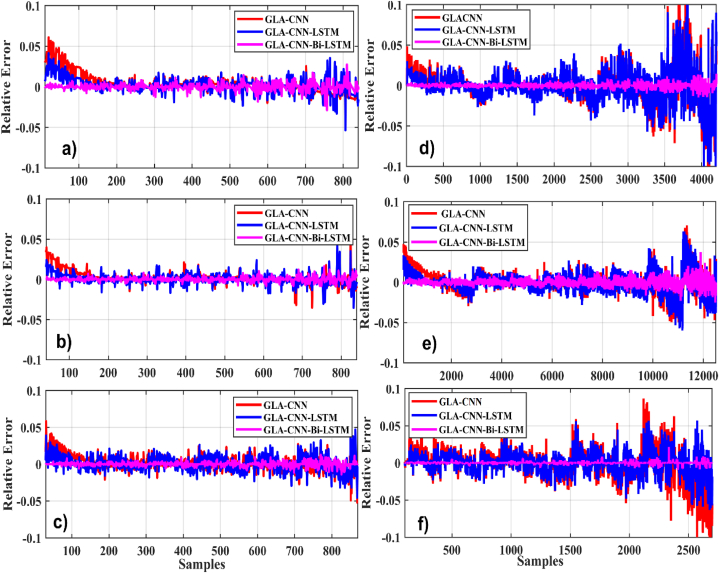
Relative error comparison for 45-degree (a) BJDST. (b) DST. (c) FUDS. (d) HWFET. (e) UDDS. (f) US06.

In this case, the temperature is set to 45 °C and the values predicted by different techniques is compared with the actual values, as given in Table 6. The values of RMSE (1.4098e-3), NMSE (2.1404e-3), MAE (0.0078) and RE (0.001) are the smallest for the proposed technique when BJDST drive cycle is implemented. In addition, when the battery is subject to DST drive cycle, the values of all parameters are the smallest for the proposed technique while the value of R-Square is close to a 100, showing that the values predicted by proposed model are more accurate as compared to the other techniques. The performance of the GLA–CNN–BiLSTM model is better than the other techniques when FUDS, HWFET, UDDS and USO6 are considered, as displayed by the relative error comparison in Fig. 13(a–f).

**Table 6:** SoC estimation evaluation at 45-degree datasets of drive cycles.

Drive Cycle	Technique	RMSE	NMSE	MAE	R^2^	RE
**BJDST**	GLA–CNN–Bi-LSTM	**1.0498e-3**	**2.1604e-3**	**0.0078**	**98.79**	**0.0001**
	GLA–CNN–LSTM	3.1750e-2	1.3306e-2	0.0207	95.22	0.0191
	GLA-CNN	7.3226e-2	7.0021e-2	0.0726	93.56	0.0398
**DST**	GLA–CNN–Bi-LSTM	**1.2344e-3**	**2.3631e-3**	**0.0009**	**99.02**	**0.0003**
	GLA–CNN–LSTM	6.1532e-3	8.4204e-3	0.0036	97.79	0.0158
	GLA-CNN	4.2710e-3	5.0014e-2	0.0401	94.12	0.0341
**FUDS**	GLA–CNN–Bi-LSTM	**2.0345e-3**	**1.8147e-3**	**0.0002**	**99.34**	**0.0014**
	GLA–CNN–LSTM	1.1819e-2	3.9058e-2	0.0057	96.24	0.0110
	GLA-CNN	8.2693e-2	5.1270e-2	0.0742	93.09	0.0289
**HWFET**	GLA–CNN–Bi-LSTM	**3.0141e-3**	**2.2785e-3**	**0.0003**	**99.16**	**0.0035**
	GLA–CNN–LSTM	9.1046e-3	8.5469e-3	0.0145	96.72	0.0722
	GLA-CNN	4.1319e-2	5.1576e-2	0.0018	94.18	0.0915
**UDDS**	GLA–CNN–Bi-LSTM	**1.3241e-3**	**1.8147e-3**	**0.0238**	**99.11**	**0.0052**
	GLA–CNN–LSTM	9.1836e-3	1.1270e-3	0.1480	96.47	0.0758
	GLA-CNN	7.2690e-2	8.6324e-2	0.7791	93.22	0.1835
**US06**	GLA–CNN–Bi-LSTM	**2.0124e-3**	**3.0975e-3**	**0.0081**	**99.06**	**0.0066**
	GLA–CNN–LSTM	8.1154e-3	9.2785e-3	0.0973	96.99	0.0449
	GLA-CNN	3.1710e-2	5.5469e-2	0.1922	94.01	0.579

**Fig. 14:**
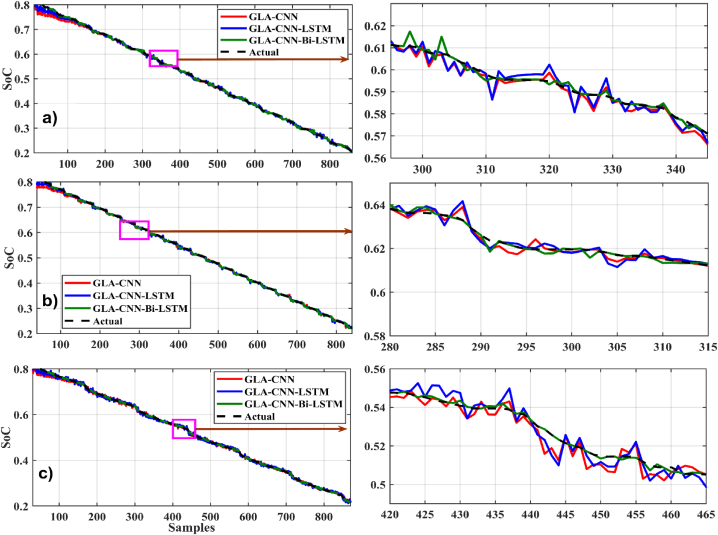
SoC estimation comparison for 45-degree (a) BJDST. (b) DST. (c) FUDS.

**Fig. 15:**
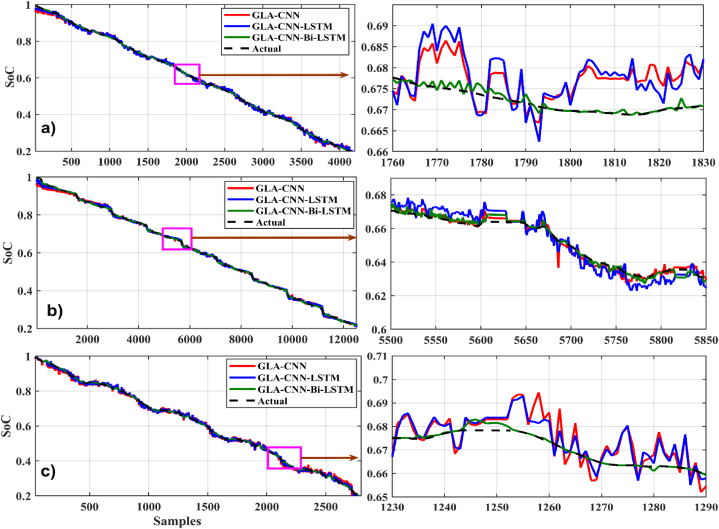
SoC estimation comparison for 45-degree. (a) HWFET. (b) UDDS. (c) US06.

Fig. 14, Fig. 15 show the graphical results of different estimation techniques at a fixed temperature of 45 °C for the selected drive cycles. In the case of BJDST, the comparison of the value predicted by different techniques with actual values shows that the GLA–CNN–BiLSTM technique has the most accurate values. The results of the BJDST, DST and FUDS drive cycles, as shown in Fig. 14, Fig. 14 (c) respectively, show that the proposed technique has least deviations compared with the actual values. When HWFET, UDDS and US06 drive cycles are considered, as given respectively in Fig. 15(a)–. (b), and Fig. 15(c), the values of the red curve (proposed technique) are close to the dashed line (the actual values). Thus, indicating the superior prediction capability of the GLA–CNN–BiLSTM model.

### 4.4 Comparative analysis

To further validate the robustness of the proposed GLA–CNN–Bi-LSTM model, it is compared with the state-of-the-art estimation techniques presented in the literature. According to Table 7, the RMSE and NMSE for SoC estimation using the GLA–CNN–Bi-LSTM technique are 0.205 % and 0.229 %, respectively. The suggested model can obtain reduced prediction errors and require less training than PSO-LSTM, BP-DNN, and Stacked Bi-LSTM. The comparative analysis further verifies that the given method can produce reliable and accurate estimation results.

**Table 7:** Comparative Analysis of GLA–CNN–Bi-LSTM with recently proposed techniques for SoC estimation.

Technique	Drive Cycles	Temperature	Error %
Proposed (GLA–CNN–Bi-LSTM)	DST, BJDST, FUDS, US06 HWFET, UDDS	$0^{o}C,25^{o}C,45^{o}C$	NMSE, 0.229 % RMSE, 0.205 %
PSO-LSTM [51]	UDDS	$25^{o}C$	MAE, 0.228 % RMSE, 0.295 %
BP-DNN [49]	DST, BJDST, FUDS, US06	$0^{o}C,25^{o}C,45^{o}C$	NMSE, 0.49 %
Stacked BiLSTM [52]	HWFET, FUDS	$25^{o}C$	RMSE, 0.86 %
LSTM-RNN [37]	DST, US06, FUDS	$0^{o}C,{\;25}^{o}C{,50}^{o}C$	RMSE, 1.3 %
PSO-TCN [40]	US06, FUDS	$0^{o}C,10^{o}C,20^{o}C,{\;25}^{o}C{{,30}^{o}C,40^{o}C,50}^{o}C$	RMSE, 1.8 % MAXE, 7.4 %
PSO-LSTM [35]	UDDS	${\;25}^{o}C$	RMSE, 0.4540 % MAE, 0.3493 %

### 4.5 Granger causality test

The Granger causality test (GCT) is an analytical speculation-based test that determines whether the anticipated distribution of a particular set of m2 time series variables, referred to as the "effect" variables, is influenced by the present and past values of a set of m1 time series variables, known as "cause," variables. A succession of events is used to model a cyclic process. In SoC prediction, the estimation solidifies the time series behavior. The core steps of Granger causality are given in Table 8.

**Table 8:** Procedure of Granger causality test.

Step #	Explanation
**Step 1:**	State the null hypothesis i.e., y(t) is not Granger-cause by x(t) **H=**$\beta_{1}=\beta_{2}=.\ldots ‥=\beta_{z}=0$
**Step 2:**	Properly choose the lag α: It is practical to choose a range of values and perform the Granger test numerous times to determine whether the outcomes are the same for various lag levels.
**Step 3:**	Use the following equations to determine if x(t) Granger-cause y(t) for different lag values: $y\left(t\right)=\sum_{i=1}^{q}\alpha_{i}y\left(t-i\right)+c_{1}+u_{1}\left(t\right)$. $y\left(t\right)=\sum_{i=1}^{z}\alpha_{i}y\left(t-i\right)+\sum_{i=1}^{z}\beta_{i}x\left(t-j\right)+c_{2}+u_{2}\left(t\right)$
**Step 4:**	Calculate F-statistic values using: $F=\frac{\left(ESS_{R}-ESS_{UR}\right)/z}{ESS_{UR}/\left(n-k\right)}$
**Hypothesis:**	If p-value < α Reject the hypothesis i.e., y(t) is Granger-cause by x(t) Else Hypothesis is true i.e., y(t) is not Granger-cause by x(t)

The results are summarized in Table 9. The statistical results clearly demonstrate the superior performance of the proposed GLA–CNN–BiLSTM method for all the considered drive cycles.

**Table 9:** Granger causality test comparison.

Drive Cycle	Technique	F-Value	SE	T statistic	P = value
BJDST	GLA–CNN–Bi-LSTM	0.6468	0.128	0.0156	0.0999
	GLA–CNN–LSTM	0.8418	0.366	0.0291	0.1073
	GLA-CNN	1.2098	1.473	0.0451	0.0942
**DST**	GLA–CNN–Bi-LSTM	0.7318	0.219	0.0078	0.1046
	GLA–CNN–LSTM	0.8785	0.503	0.0382	0.0978
	GLA-CNN	1.2138	1.012	0.0417	0.0914
**FUDS**	GLA–CNN–Bi-LSTM	0.6515	0.029	0.0123	0.1002
	GLA–CNN–LSTM	0.8293	0.139	0.0318	0.1045
	GLA-CNN	1.3037	1.253	0.0433	0.0903
**HWFET**	GLA–CNN–Bi-LSTM	0.6238	0.180	0.0199	0.1087
	GLA–CNN–LSTM	0.8319	1.411	0.0366	0.0981
	GLA-CNN	1.3037	1.253	0.0433	0.0903

**UDDS**	GLA–CNN–Bi-LSTM	0.6352	0.141	0.0132	0.1008
	GLA–CNN–LSTM	0.8116	1.481	0.0378	0.0989
	GLA-CNN	1.3191	1.218	0.0401	0.0926
**US06**	GLA–CNN–Bi-LSTM	0.6822	0.137	0.0198	0.1861
	GLA–CNN–LSTM	0.8795	1.499	0.0403	0.0159
	GLA-CNN	1.3184	1.201	0.0465	0.0843

## 5 Conclusions

In this paper, we introduced a groundbreaking hybrid CNN–Bi-LSTM architecture designed for accurate and efficient State of Charge (SoC) estimation of lithium-ion batteries. A key innovation of this work is the use of the Group Learning Algorithm (GLA) to optimally tune the hyperparameters of the CNN–Bi-LSTM network. This strategic tuning significantly enhances the predictability and stability of the proposed architecture, providing a robust solution that reduces the complexities associated with manually setting hyperparameters, which is a common limitation in traditional approaches. The integration of GLA with the CNN–Bi-LSTM model represents a novel approach that alleviates the computational burdens typically encountered in deep learning models. This synergy not only improves the efficiency of the estimation process but also enhances the model's ability to adapt to diverse operational conditions without the risk of overfitting or underfitting. Performance evaluations using a variety of metrics such as Mean Absolute Error (MAE), Root Mean Squared Error (RMSE), Relative Error (RE), and the Granger causality test have demonstrated the model's superior accuracy and reliability. The model achieved remarkably low error rates—0.229 % NMSE, 0.205 % RMSE, 0.103 % MAE, and 0.01 % RE—outperforming contemporary state-of-the-art models. This confirms not only the effectiveness of the hybrid model in capturing the intricate dynamics of battery behavior but also its potential in real-world applications. Furthermore, the Granger causality test provided additional validation of the model's capability to accurately forecast SoC based on historical and current data inputs, further emphasizing its analytical prowess. Extensive comparative experiments conducted across various temperatures and six distinct EV drive cycles reinforced that the proposed model offers unparalleled SoC estimation precision and adaptability.

## Availability of data and access

The data used in this work will be made available on request.

## CRediT authorship contribution statement

**Muhammad Kamran Khan:** Methodology, Formal analysis, Data curation, Conceptualization. **Mohamad Abou Houran:** Writing – original draft, Visualization, Software, Investigation, Formal analysis. **Kimmo Kauhaniemi:** Validation, Formal analysis. **Muhammad Hamza Zafar:** Writing – review & editing, Supervision, Software, Methodology. **Majad Mansoor:** Writing – review & editing, Writing – original draft, Validation, Project administration, Methodology. **Saad Rashid:** Software, Resources, Formal analysis.

## Declaration of competing interest

The authors declare that they have no known competing financial interests or personal relationships that could have appeared to influence the work reported in this paper.
